# The Diversity of the Linguofacial Trunk

**DOI:** 10.3390/medicina60020291

**Published:** 2024-02-08

**Authors:** Cătălin Constantin Dumitru, Alexandra Diana Vrapciu, Mugurel Constantin Rusu

**Affiliations:** 1 Division of Anatomy, Faculty of Dentistry, “Carol Davila” University of Medicine and Pharmacy, 050474 Bucharest, Romania; catalin-constantin.dumitru@drd.umfcd.ro (C.C.D.); mugurel.rusu@umfcd.ro (M.C.R.); 2 University Emergency Hospital Bucharest, 050098 Bucharest, Romania

**Keywords:** carotid artery, lingual artery, facial artery, hyoid bone, gonial angle, anatomic variation

## Abstract

*Background and Objectives*: Typically, the external carotid artery (ECA) sends off separate anterior branches: the superior thyroid, lingual, and facial arteries. These could, however, form common trunks: thyrolinguofacial, linguofacial (LFT), or thyrolingual. Although known, the LFT variant was poorly detailed previously, and most authors just counted the variant. We aimed to demonstrate the individual anatomical possibilities of the LFT on a case-by-case basis. *Materials and Methods*: 150 archived angioCT files were used. After applying inclusion and exclusion criteria, 147 files of 86 males and 61 females were kept for this study. *Results*: In 34/147 cases, LFTs were found (23.12%). Bilateral LFTs were found in 13/34 cases (38.24%) and unilateral LFTs in 21/34 (61.76%) cases. Forty-seven LFTs were thus identified and further studied for different variables. Regarding the vertical topography of LFT origin, type 1a (suprahyoid and infragonial) was found in 28 LFTs (59.57%), type 1b (suprahyoid and gonial) was found in eight LFTs (17.02%), type 3 (suprahyoid and supragonial) was found in two LFTs (4.25%), type 2 (hyoid level of origin) in eight LFTs (17.02%), and type 3 (infrahyoid origin) in just one LFT (2.12%). Types of the initial course of the LFT were determined: type I, ascending, was found in 22/47 LFTs; type II, descending, in 12/47 LFTs; and type III, transverse, in 13/47 LFTs. Regarding the orientation of the first loop of the LFT, 23/47 LFTs had no loop, 4/47 had anterior loops, 1/47 had a posterior loop, 5/47 had superior loops, 5/47 had inferior loops, and 9/47 had medial loops. The position of the LFT relative to the ECA was classified as medial, anterior, or antero-medial. An amount of 12/47 LFTs were anterior to the ECA, 22/47 were antero-medial, 10/47 were medial, 2/47 were inferior, and 1/47 was lateral. Regarding their general morphology, 23/47 LFTs had a rectilinear course, 22/47 had loops, and 2/47 were coiled. A case-by-case presentation of results further demonstrated the diversity of the LFT. *Conclusions*: In conclusion, the morphology and topography of the LFT are individually specific and unpredictable. It can be anticipated case-by-case by surgeons on CT or MR angiograms.

## 1. Introduction

In typical anatomy, the external carotid artery (ECA) gives off distinctive collateral branches: the superior thyroid (STA), lingual (LA), facial (FA), ascending pharyngeal (APA), occipital (OA) and posterior auricular (PAA) arteries [1]. Successive arteries could leave the ECA as common trunks, such as thyrolinguofacial, thyrolingual, thyrolinguolaryngeal, linguofacial, or occipitoauricular trunks [2,3,4]. Knowledge of the variations in the branching pattern of the ECA is essential for surgeons, as the planning and execution of surgery is purely based on the anatomical pattern of the structures in the neck [5].

The linguofacial trunk (LFT) is commonly regarded and reported as a unique anatomical variant [2,6,7,8,9,10,11,12,13]. This is true if one observes it just as a common trunk of origin of the LA and FA. However, the morphology and topography of the LFT should be individually specific. The morphometric characteristics of the LFT have been only rarely described [14].

This study was, therefore, aimed at demonstrating on a case-by-case basis the individual anatomical possibilities of the LFT. The incidence of the LFT and the heterogeneous morphological possibilities were established.

## 2. Materials and Methods

There were 150 archived angioCT files used. Inclusion criteria were the age of the subjects (>18 years), adequate quality of the scans, and no pathologic processes distorting the neck anatomy. Exclusion criteria were pathological processes distorting the arterial anatomy and degraded or incomplete scans. After applying inclusion and exclusion criteria, 147 files of 86 males and 61 females were kept for this study.

The first two authors documented the anatomical variants using the Horos 4.0.1 for Apple Silicon software (Horos Project), as in previous studies [15,16,17]. The research was conducted ethically following The Code of Ethics of the World Medical Association (Declaration of Helsinki). The subjects gave their informed consent for inclusion before participating in the study, and the responsible authorities (affiliation 2) approved the study (approval no.10540/16.02.2022).

Different variables of the LFT were tracked as follows: Types 1–3 of the vertical topography of LFT origin from the ECA: type 1, origin superior to the hyoid bone (subtype 1a—inferior to gonial angle, subtype 1b—gonial level, and type 1c—superior to gonial angle); type 2, origin of the LFT at the level of the hyoid bone; and type 3, infrahyoid origin of the LFT. Types I–III of the initial course of the LFT: type I—ascending, type II—descending, and type III—transverse. The orientation of the first loop of the LFT has been classified as follows: S—superior orientation, I—inferior orientation, M—medial orientation, A—anterior orientation, P—posterior orientation, and NL—no initial loop. The position of the LFT relative to the ECA was classified as medial, anterior, or antero-medial. The general LFT morphology was systematized: R—rectilinear LFT, L—general loop, and C—arterial coil.

## 3. Results

Of the 147 cases, in 34 cases, 16 males and 18 females were found to have LFTs (23.12%). There were 13 cases (38.24%) with bilateral LFTs: six males and seven females. A left LFT was found in 13 cases (seven males, six females) (38.24%). A right LFT was found in eight cases (three males, five females) (23.53%).

Forty-seven LFTs were thus identified and further studied for several other variables: the vertical topography of the LFT origin relative to the hyoid, the initial course of the LFT, the convexity of the first TLF loop or rectilinear course, the position relative to the ECA, and the overall morphology (loop/coil/rectilinear).

### 3.1. The Vertical Topography of the LFT

On the right side, 21 LFTs were found. Nine of these right LTFs were found in males and 12 In females. In males, the vertical topography of the LFT origin was of type 1a (origin between the hyoid and the gonial angle) in six cases and type 2 (origin at the level of the hyoid bone) in three cases. In females, type 1a was found in eight cases, type 1b (gonial level of the LFT origin) in two cases, and type 2 in two other cases.

On the left side, 26 LFTs were found. Thirteen of these were in males and thirteen in females. The vertical topography of those left LFTs was as follows. In males, type 1a was found in seven instances, type 1b in three cases, type 1c (supragonial origin of the LFT) in one case, and type 2 in two cases. In six female cases, the left LFT was of type 1a in six cases, type 1b in three instances, type 1c in two cases, type 2 in one case, and type 3 (infrahyoid origin of the LFT) in one case.

Therefore, in the general lot of the 47 LFTs that were found, there were 28 LFTs of type 1a (59.57%), eight LFTs of type 1b (17.02%), two LFTs of type 1c (4.25%), eight LFTs of type 2 (17.02%), and just one LFT of type 3 (2.12%).

### 3.2. The Initial Course of the LFT

We found the following distribution regarding the initial direction of the LFT (types I–III). In males, the nine right LFTs were of type I (initially ascending) in seven cases and of type III (initial transverse course) in two instances; no right LFT was of type II (initially descending). The 13 left LFTs found in males were of type I in eight cases, type II in three cases, and type III in two cases. The twelve right LFTs in females were of type I in four cases, type II in four cases, and type III in four cases. On the left side in females, three LFTs of type I, five LFTs of type II, and five LFTs of type III were found. Therefore, of 47 LFTs, 22 (46.8%) were of type I, 12 (25.53%) were of type II, and 13 (27.65%) were of type III.

### 3.3. The Orientation of the First Loop of the LFT

Regarding the orientation of the first loop of the LFT, nine right LFTs in males were found: one case with an anteriorly oriented loop, one with a posterior orientation of the loop, two cases with the loop oriented medially, and five cases without an initial loop. In the 12 female cases with right LFTs, one had an anterior orientation, two were oriented inferiorly, two were oriented medially, and in seven cases, no loop was found. On the left side, the 13 LFTs in males were oriented anteriorly in one case, superiorly in four instances, medially in three cases, and in five cases, there were no loops. In the 13 left LFTs in females, we found one oriented anteriorly, one oriented superiorly, three with inferior orientation, and two oriented medially, and there were no such loops in the remaining six cases. In males, on the right side, 5/9 LFTs showed no loops; and on the left, 5/13 LFTs showed no loops. In females on the right, 7/12 LFTs showed no loops; and on the left, 6/13 LFTs showed no loops. In both sexes, we found no right LFT with a superior loop and no left LFT with a posterior loop.

Therefore, 23/47 LFTs had no loop, 4/47 had anterior loops, 1/47 had a posterior loop, 5/47 had superior loops, 5/47 had inferior loops, and 9/47 had medial loops.

### 3.4. The Position of the LFT Relative to the ECA

Regarding the position of the LFT relative to the ECA in males, three right and four left LFTs were anterior to the ECA, five right and nine left LFTs were antero-medial to the ECA, and one right LFT was medial to the ECA. In males, no LFTs were found inferior or lateral to the ECA. In females, three right and two left LFTs were anterior to the ECA, four right and four left LFTs were antero-medial to the ECA, five right and four left LFTs were medial to the ECA, two left LFTs were inferior to the ECA, and one left LFT crossed the ECA laterally.

Therefore, 12/47 LFTs were anterior to the ECA, 22/47 were antero-medial, 10/47 were medial, 2/47 were inferior, and 1/47 was lateral.

### 3.5. The General LFT Morphology

Regarding the general morphology of the LFT in males, five right and five left LFTs were rectilinear, four right and seven left LFTs were looped, and one left LFT was coiled. In females, seven right and six left LFTs were rectilinear, four right and seven left LFTs were looped, and one right LFT was coiled. So, only in two cases, one female and one male, was the LFT coiled. So, 23/47 LFTs had a rectilinear course, 22/47 had loops, and 2/47 were coiled.

### 3.6. Unilateral Left Linguofacial Trunks

Thirteen unilateral left LFTs were found.

**Figure 1:**
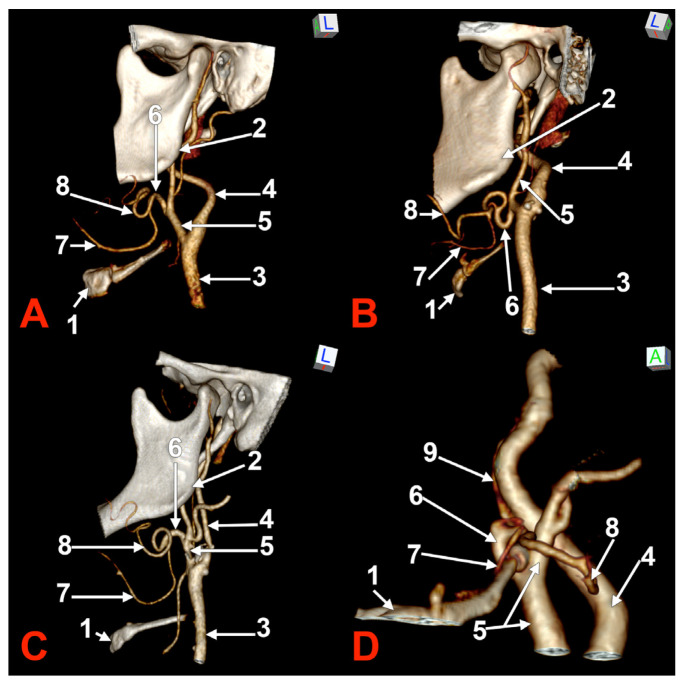
Morphological variants of the left unilateral linguofacial trunk. Computed tomography angiograms. Three-dimensional volume renderings. Infero-lateral (**A**–**C**) and antero-lateral (**D**) views. 1. hyoid body; 2. gonial angle; 3. common carotid artery; 4. internal carotid artery; 5. external carotid artery; 6. linguofacial trunk; 7. lingual artery; 8. facial artery; and 9. ascending pharyngeal artery.

In case #1 (male) a left LFT left from the medial side of the ECA was found (Figure 1A) at 1.8 cm postero-superior to the tip of the greater horn of the hyoid bone, therefore in the interval between the hyoid and the gonial angle. That left LFT had an initial loop oriented superiorly and was dividing into the LA and FA at 0.66 cm medially to the ECA.

In case #2 (female), the LFT left from the left ECA was found at 0.37 cm inferior to the gonial angle and 1.29 cm postero-superior to the tip of the greater horn of the hyoid bone, therefore in the interval between the hyoid and the gonial angle. That LFT was initially descending, and the LFT had a general loop oriented inferiorly (Figure 1B).

In case #3 (male), a left LFT originated from the ECA at 3.54 cm postero-superior to the tip of the greater horn of the hyoid bone, in the interval between the hyoid and the gonial angle. It ascended medially to the ECA and described a superiorly convex loop (Figure 1C).

In case #4 (female), the LFT left originated from the medial side of the left ECA immediately inferior to the tip of the greater horn of the hyoid bone and further continued medially to it. Therefore, the tip of the greater horn was placed between the ECA on the outer side and the LFT on the inner side (Figure 1D). Although the LFT was initially ascending, it had an incomplete inferior loop posterior to the pharyngeal wall. Due to that course of the LFT, the LA and FA coursed transversally from medial to lateral and crossed anteriorly the superior thyroid artery and the ECA.

**Figure 2:**
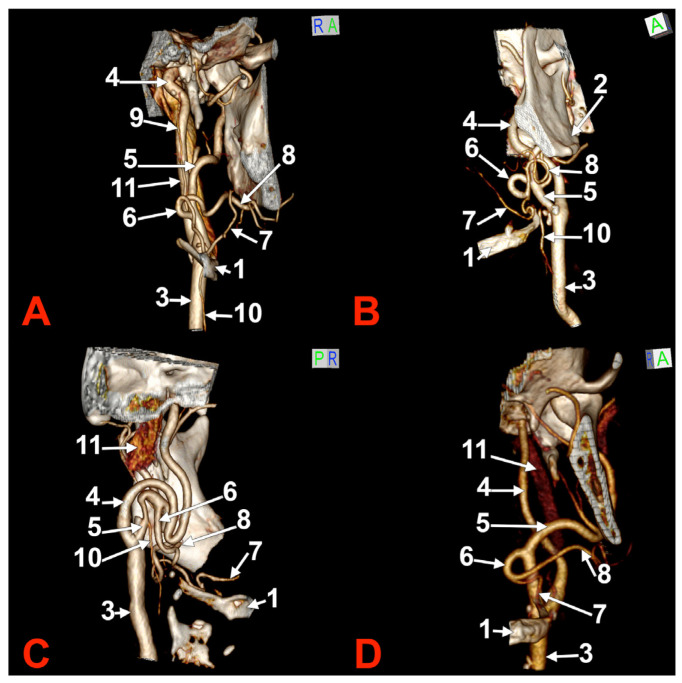
Morphological variants of the left unilateral linguofacial trunk. Computed tomography angiograms. Three-dimensional volume renderings. Anterior (**A**,**D**), antero-lateral, (**B**) and antero-medial (**C**) views. 1. hyoid body; 2. gonial angle; 3. common carotid artery; 4. internal carotid artery; 5. external carotid artery; 6. linguofacial trunk; 7. lingual artery; 8. facial artery; 9. ascending pharyngeal artery; 10. superior thyroid artery; and 11. internal jugular vein.

In case #5 (male), the origin of the LFT from the left ECA was at 1.19 cm postero-superior to the tip of the greater horn of the hyoid bone but at 2.29 cm deep and not inferior to the gonial angle (Figure 2A). That LFT divided into the FA and LA at 0.49 cm antero-medially to the ECA.

In case #6 (male), a large and coiled left LFT (Figure 2B) was found that originated from the posterior side of the ECA distally to the occipital artery and at 1.15 cm postero-superior to the tip of the greater horn of the hyoid bone. While the caliber of the ECA was 0.44 cm, that of the LFT was 0.34 cm.

In case #7 (female), the carotid bifurcations were placed superior to the gonial angle, and both the ECA and the internal carotid artery (ICA) were looped deep to the gonial angles. Therefore, the ECA branches were longer than usual. A long left LFT descended at 1.11 cm deep to the gonial angle. The arterial morphology of the ECA, ICA, and LFT was that of an arterial labyrinth (Figure 2C). Due to the loop of the ECA, the LFT appeared to be an inferior branch of it. The origin of the LFT was 2.82 cm above the greater horn of the hyoid bone. The course of the LFT was rectilinear. At 1.34 cm, postero-supero-lateral to the tip of the greater horn, the LFT divided into the LA and FA.

In case #8 (male), the origin of the LFT from the left ECA was deep to the gonial angle, 1.67 cm postero-superior to the tip of the greater horn of the hyoid bone. It had a medially oriented loop (Figure 2D).

**Figure 3:**
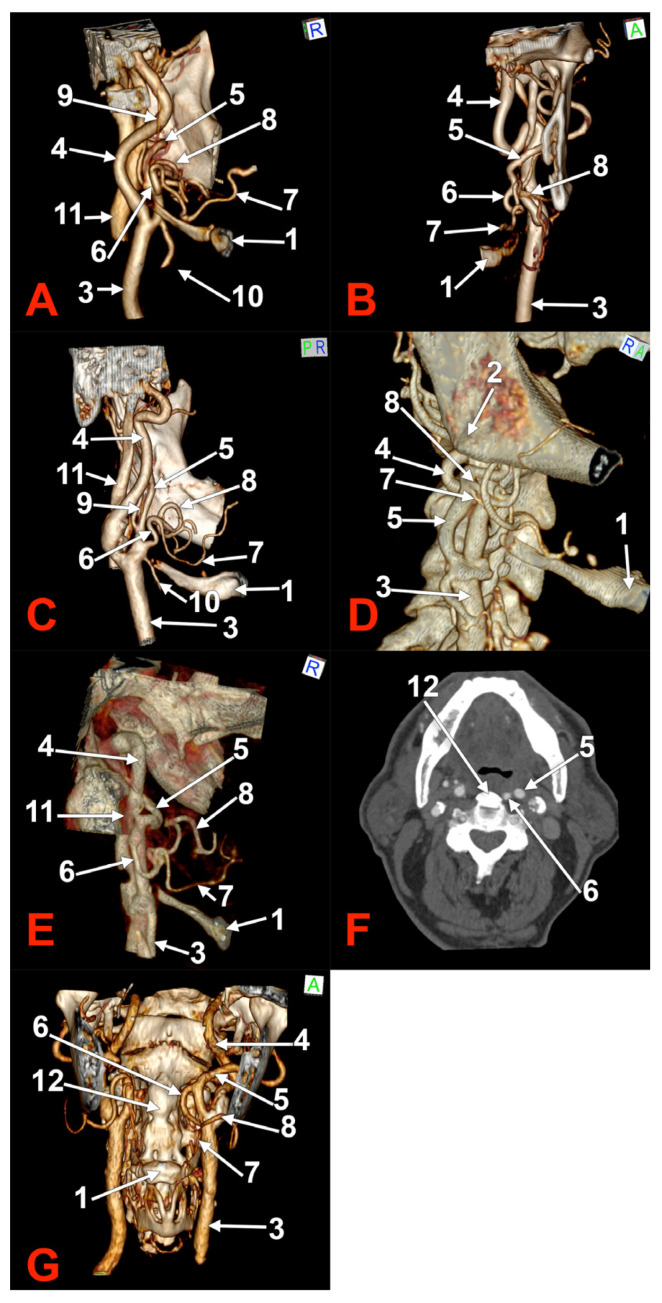
Morphological variants of unilateral left linguofacial trunks. Computed tomography angiograms. Three-dimensional volume renderings. Anterior (**B**,**C**,**G**), right lateral (**D**), and medial (**A**,**C**,**E**) views. Axial slice (**F**). 1. hyoid body; 2. gonial angle; 3. common carotid artery; 4. internal carotid artery; 5. external carotid artery; 6. linguofacial trunk; 7. lingual artery; 8. facial artery; 9. ascending pharyngeal artery; 10. superior thyroid artery. 11. internal jugular vein; and 12. ossified anterior longitudinal ligament.

In case #9 (female), the left carotid bifurcation (CB) was immediately infero-lateral to the greater horn of the hyoid bone, and the initial segment of the ECA was applied on the lateral side of the greater horn (Figure 3A). The LFT originated from the left ECA at 0.22 cm above the tip of the greater horn. It was profound to the gonial angle. It was initially ascending and had a superior loop. It first gave off the LA, then the FA. Therefore, the LA crossed over the FA.

In case #10 (female), the left submandibular gland descended over the hyoid bone’s greater horn, and the ECA ascended deep to the gland. A 0.93 cm long LFT originated from the medial side of the left ECA, deep to the gonial angle (Figure 3B). At the same level, an occipitoauricular trunk formed from the lateral side of the ECA. The origin of the LFT was at 1.74 cm postero-superior to the tip of the greater horn of the hyoid bone. It had an initial descending course and looped inferiorly.

In case #11 (male), an LFT was found originating from the anterior side of the left ECA in the interval between the hyoid and the gonial angle, at 0.53 cm supero-lateral to the tip of the greater horn of the hyoid bone (Figure 3C). That LFT had a tortuous ascending course and reached the inferior margin of the mandible, where it divided into the LA and FA.

In case #12 (female) a left LFT was found, and, on the right side, adjacent origins of the LA and FA from the ECA (Figure 3D). The right LA and FA left the ECA in this order at 0.91 cm postero-superior to the tip of the right greater hyoid horn, therefore, in the interval between the hyoid and the gonial angle. The left LFT originated from the ECA at 1.59 cm postero-superior to the tip of the greater hyoid horn, deep to the gonial angle. It further descended and was applied on the medial side of the ECA to reach 0.27 cm superior to the tip of the greater hyoid horn. It looped inferiorly and ascended to further divide into the LA and FA at 1.01 cm superior to the greater horn (Figure 3E).

In case #13 (male), a unilateral LFT was found on the left side. It was retropharyngeal, at 2.44 mm lateral to an ossified anterior longitudinal ligament (Figure 3F). The origin of that LFT was at 2.88 cm supero-medial to the tip of the greater horn of the hyoid bone, deep to the gonial angle. It had a rectilinear descending retropharyngeal course at 0.37 cm medial to the ECA at the level of the third cervical vertebra (Figure 3G). The LFT divided into the LA and FA at the C3/C4 intervertebral disc level, those two arteries being medial to the CB and the superior thyroid artery.

### 3.7. Unilateral Right Linguofacial Trunks

Eight unilateral right LFTs were found.

**Figure 4:**
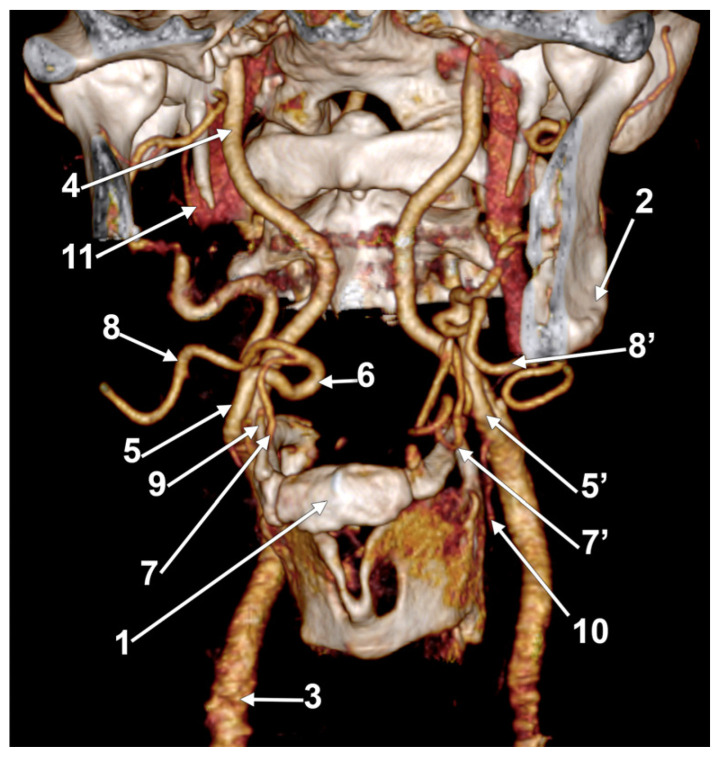
Right linguofacial trunk. Computed tomography angiogram. Three-dimensional volume rendering. Antero-inferior view. 1. hyoid body; 2. gonial angle; 3. right common carotid artery; 4. right internal carotid artery; 5, 5’. external carotid arteries; 6. linguofacial trunk; 7, 7’. lingual arteries; 8, 8’. facial arteries; 9. ossified ceratohyal; 10. superior thyroid arteryș and 11. internal jugular vein.

In case #14 (male), the right ECA was applied on the lateral side of the greater hyoid horn. On that side, the lesser hyoid horn was 16.5 mm long, suggesting a distal ossification of the ceratohyal. A right LFT was found leaving from the ECA (Figure 4) at 0.83 cm superior to the lesser hyoid horn and 1.89 cm antero-superior to the tip of the greater hyoid horn. This latter was contacting the superior horn of the thyroid cartilage (absent lateral thyrohyoid ligament). The LFT coursed initially transversally and described a medial loop crossing anteriorly to the ICA. Anterior to the ICA’s inner side, the LFT divided into the LA and FA. The right LA further crossed the LFT anteriorly to reach the greater hyoid horn. Therefore, the LFT coursed between the ICA posteriorly and the LA anteriorly.

**Figure 5:**
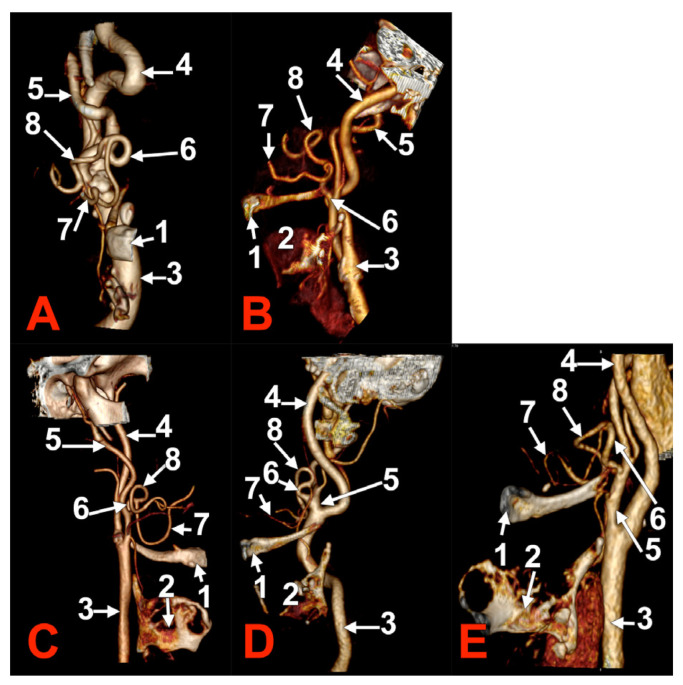
Morphological variants of right unilateral linguofacial trunks. Computed tomography angiograms. Three-dimensional volume renderings. Anterior (**A**), antero-medial (**B**,**D**,**E**), and medial (**C**) views. 1. hyoid body; 2. thyroid cartilage; 3. common carotid artery; 4. internal carotid artery; 5. external carotid artery; 6. linguofacial trunk; 7. lingual artery; and 8. facial artery.

In case #15 (female), a right LFT originated from the ECA deep to the gonial angle, at 1.34 cm postero-superior to the tip of the greater horn of the hyoid bone. It coursed transversally medially and described a complete coil on the medial side of the ECA. The coil divided into the LA and FA that crossed the origin of the LFT anteriorly (Figure 5A).

In case #16 (female), the right LFT origin from the ECA was in the interval between the hyoid and the gonial angle. It had a short descending rectilinear course and was divided into the LA and FA at 0.76 cm superior to the tip of the greater horn.

In female case #17, the CBs were located inferiorly at the level of the cricoid cartilage. Therefore, the right LFT originated from the anterior side of the ECA at 0.84 cm postero-inferior to the tip of the greater horn of the hyoid bone (Figure 5B). On the outer side of the LFT origin was an anterior loop of the ICA. That LFT ascended 0.92 cm anteriorly to the ECA, crossing the greater horn of the hyoid bone laterally.

In case #18 (male), a right LFT originated from the ECA at 1.45 cm superior to the greater hyoid horn in the interval between the hyoid and the gonial angle (Figure 5C). It ascended on the medial side of the ECA and looped posteriorly.

In case #19 (female), the origin of the right LFT was at 1.01 cm superior to the tip of the greater hyoid horn. It described an anterior loop (Figure 5D).

In case #20 (male), the right LFT originated from the ECA at the level of the hyoid bone, at 1.8 mm postero-lateral to its tip (Figure 5E). It ascended and crossed the ECA anteriorly.

**Figure 6:**
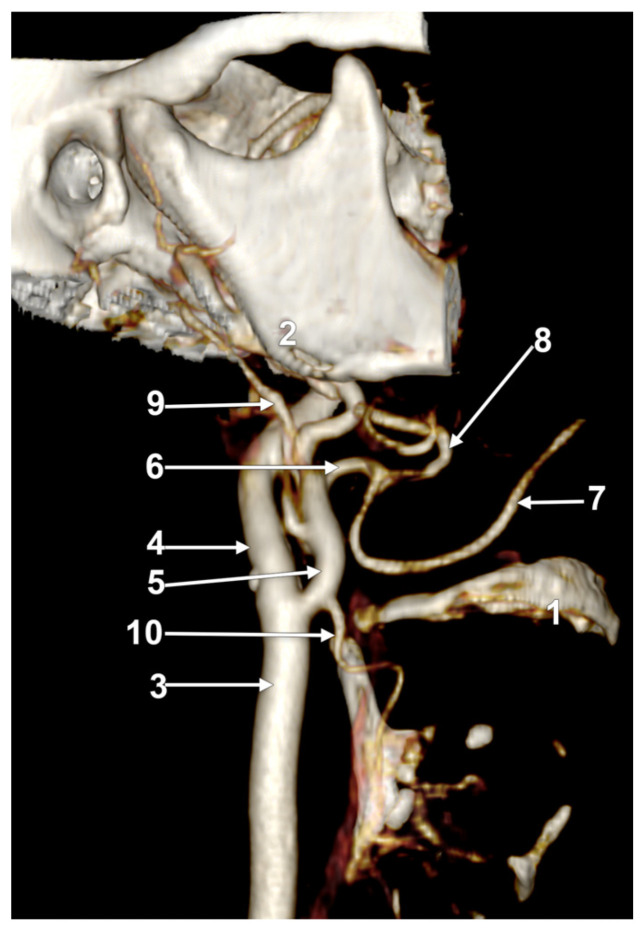
Right linguofacial trunk, CT angiogram. Three-dimensional volume rendering. Right lateral view. 1. hyoid body; 2. gonial angle; 3. common carotid artery; 4. internal carotid artery; 5. external carotid artery; 6. linguofacial trunk; 7. lingual artery; 8. facial artery; 9. occipital arteryș and 10. superior thyroid artery.

In case #21 (female), the right LFT originated from the ECA at 2.22 cm postero-supero-lateral to the greater horn of the hyoid bone but inferior to the gonial angle (Figure 6). It had a transverse rectilinear course. It divided into the LA and FA at 1.91 cm superior to the greater horn of the hyoid bone.

### 3.8. Bilateral Linguofacial Trunks

In thirteen cases, bilateral linguofacial trunks were found.

**Figure 7:**
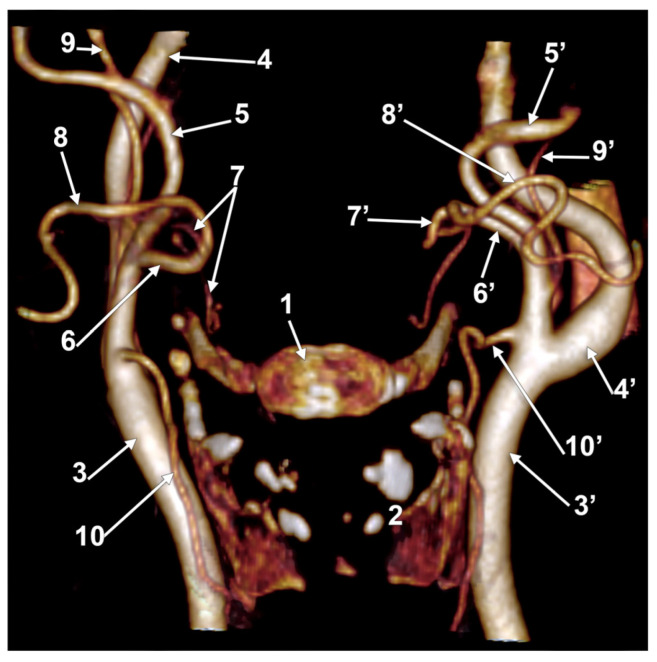
Bilateral linguofacial trunk. Computed tomography angiogram. Three-dimensional volume rendering. Anterior view. 1. hyoid body; 2. thyroid cartilage; 3, 3’. common carotid arteries; 4, 4’. internal carotid arteries; 5, 5’. external carotid arteries; 6, 6’. linguofacial trunks; 7, 7’. lingual arteries; 8, 8’. facial arteries; 9, 9’. occipital arteries; and 10, 10’. superior thyroid arteries.

In case #22 (female), the LFTs were morphologically and topographically bilaterally asymmetrical (Figure 7). The right LFT left the anterior side of the ECA in the interval between the hyoid and gonial angle, at 1.4 cm postero-superior to the tip of the greater horn. It coursed anteriorly and reached 0.52 cm superior to the tip of the greater horn to divide into the LA and FA. The left LFT origin was on the medial side of the ECA, at 1.18 cm postero-supero-lateral to the greater hyoid horn, and had a medial rectilinear course to divide into the LA and FA at 1.28 cm postero-superior to the tip of the greater horn of the hyoid bone.

**Figure 8:**
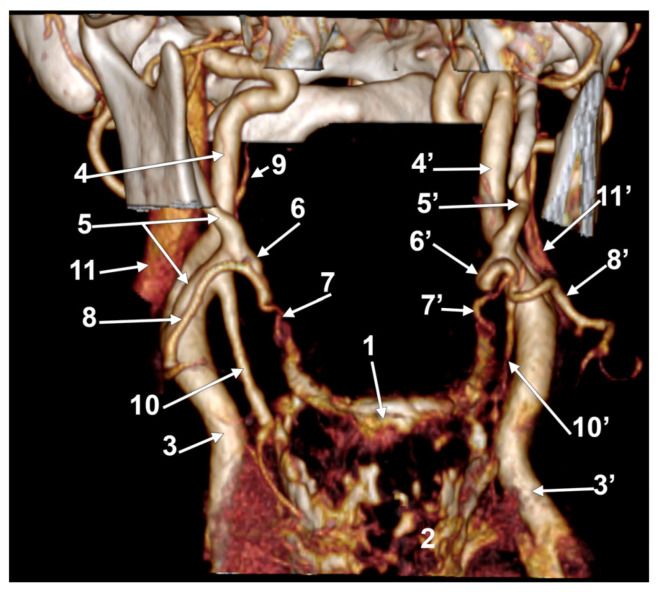
Bilateral linguofacial trunk. Computed tomography angiogram. Three-dimensional volume rendering. Anterior view. 1. hyoid body; 2. thyroid cartilage; 3, 3’. common carotid arteries; 4, 4’. internal carotid arteries; 5, 5’. external carotid arteries; 6, 6’. linguofacial trunks; 7, 7’. lingual arteries; 8, 8’. facial arteries; 9. ascending pharyngeal artery; 10, 10’. superior thyroid arteries; and 11, 11’. internal jugular veins.

In case #23 (female), there was a degree of bilateral symmetry of the LFTs (Figure 8). The CBs, origins of the superior thyroid arteries, and the LFTs were located beneath the angle of the mandible on each side. The right LFT left the ECA at 1.48 cm postero-supero-lateral to the tip of the greater horn of the hyoid bone and at 0.67 cm superior to the origin of the superior thyroid artery. It coursed antero-inferiorly, without a loop, and divided into the LA and FA deep to the submandibular gland, at 1.49 cm supero-lateral to the greater horn of the hyoid bone. The left LFT origin from the ECA was 1.85 cm postero-supero-lateral to the tip of the greater hyoid horn. It initially coursed antero-inferiorly and divided into the LA and FA at 1.24 cm supero-lateral to the greater horn.

**Figure 9:**
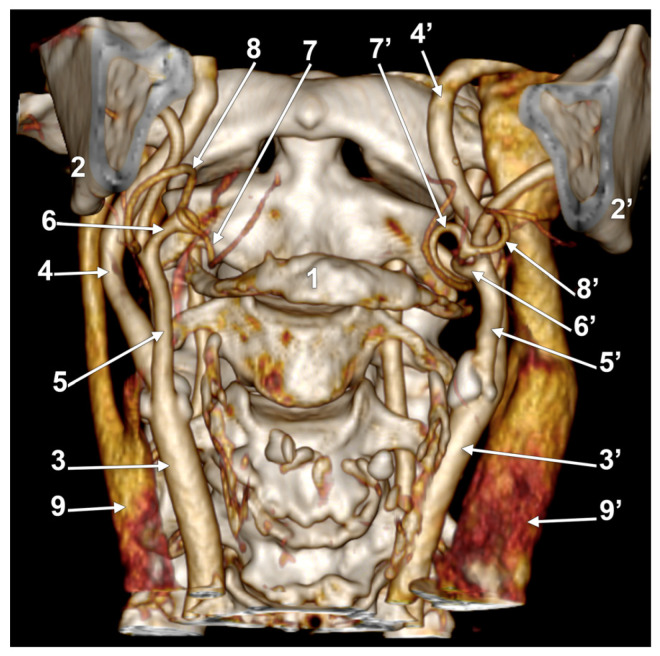
Bilateral linguofacial trunk. Computed tomography angiogram. Three-dimensional volume rendering. Anterior view. 1. hyoid body; 2, 2’. gonial angles; 3, 3’. common carotid arteries; 4, 4’. internal carotid arteries; 5, 5’. external carotid arteries; 6, 6’. linguofacial trunks; 7, 7’. lingual arteries; 8, 8’. facial arteries; and 9, 9’. internal jugular veins.

Case #24 (female) had bilateral LFTs originating each from the ECA in the interval between the hyoid bone and the gonial angle (Figure 9). The right LFT origin was at 0.79 cm postero-supero-lateral to the tip of the greater horn of the hyoid bone. It had a rectilinear antero-superior course. It was divided into the LA and FA deep to the submandibular gland at 0.58 cm, superior to the tip of the greater hyoid horn. The left LFT origin was 0.63 cm postero-superior to the tip of the greater horn. It had a rectilinear anterior course and was divided into the LA and FA at 0.67 cm superior to the greater horn’s tip.

**Figure 10:**
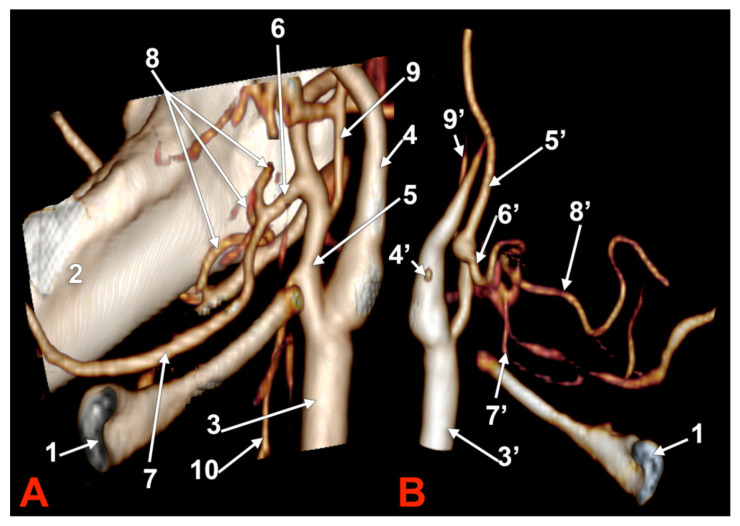
Bilateral linguofacial trunk. Computed tomography angiogram. Three-dimensional volume rendering. Antero-medial views, right side (**A**) and left side (**B**). 1. hyoid body; 2. mandible; 3, 3’. common carotid arteries; 4, 4’. internal carotid arteries; 5, 5’. external carotid arteries; 6, 6’. linguofacial trunks; 7, 7’. lingual arteries; 8, 8’. facial arteries; 9, 9’. occipital arteries; and 10. superior thyroid artery.

In case #25 (female), the LFTs were relatively bilaterally symmetrical. The right LFT (Figure 10A) left the ECA at 1.29 cm postero-supero-lateral to the tip of the greater horn of the hyoid bone, deep to the gonial angle. It had a rectilinear antero-inferior course and, at 1.08 cm superior to the tip of the greater horn, divided into the LA and FA. The left LFT origin was at 1.69 cm postero-supero-lateral to the tip of the greater hyoid horn, deep to the gonial angle (Figure 10B). It descended antero-inferiorly and at 0.87 cm superior to the greater horn’s tip, it divided into the LA and FA.

**Figure 11:**
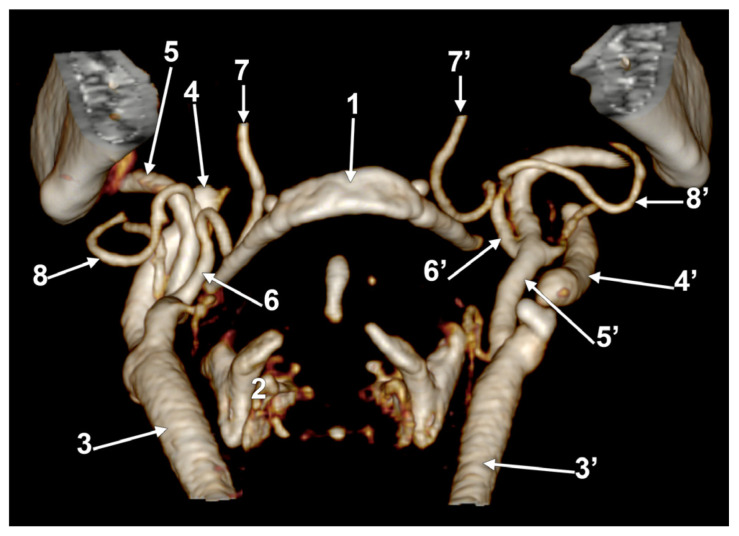
Bilateral linguofacial trunk. Computed tomography angiogram. Three-dimensional volume rendering. Antero-inferior view. 1. hyoid body; 2. thyroid cartilage; 3, 3’. common carotid arteries; 4, 4’. internal carotid arteries; 5, 5’. external carotid arteries; 6, 6’. linguofacial trunks; 7, 7’. lingual arteries; and 8, 8’. facial arteries.

In case #26 (female), the CBs were located at the level of the superior margin of the thyroid cartilage, but the right one was sagittal, while the left one was coronal. Therefore, the course of the bilateral LFTs differed. Both LFTs had a hyoid level of origin (Figure 11). The right one originated from the anterior side of the ECA, at 1.2 cm postero-superior to the tip of the greater hyoid’s horn. It coursed anteriorly, without a loop, reaching 0.49 cm superior to the tip of the greater horn, deep to the submandibular gland, where it divided. The left LFT origin was from the medial side of the ECA, at 0.54 cm postero-lateral to the tip of the greater horn. It ascended supero-medially and divided into the LA and FA at 0.87 cm postero-superior to the tip of the greater horn.

**Figure 12:**
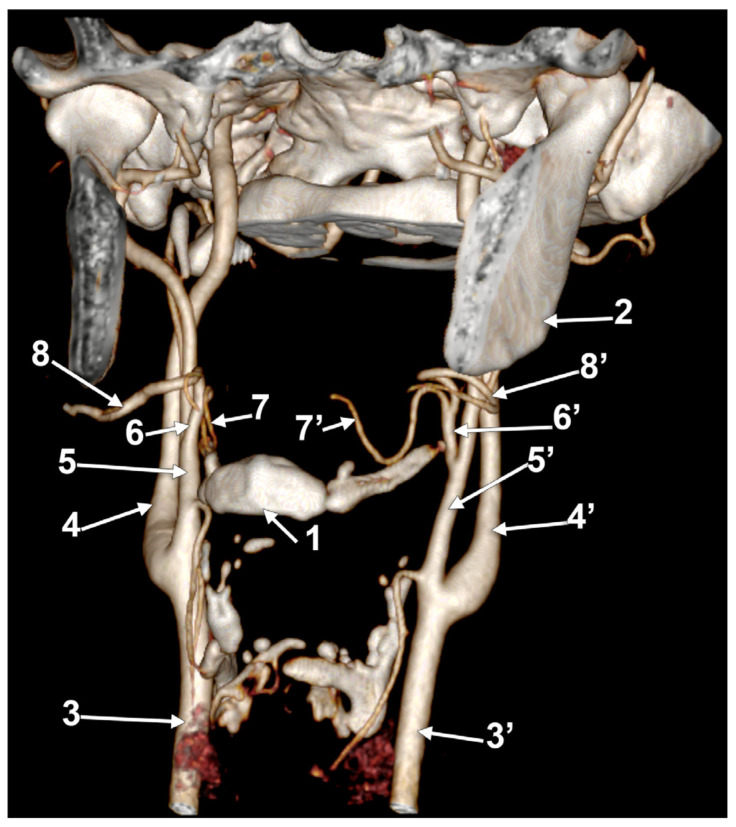
Bilateral linguofacial trunk. Computed tomography angiogram. Three-dimensional volume rendering. Antero-inferior view. 1. hyoid body; 2. gonial angle; 3, 3’. common carotid arteries; 4, 4’. internal carotid arteries; 5, 5’. external carotid arteries; 6, 6’. linguofacial trunks; 7, 7’. lingual arteries; and 8, 8’. facial arteries.

In case #27 (male), there were bilateral LFTs with low origins (Figure 12). The right one’s origin from the ECA was immediately postero-lateral (0.15 cm) to the tip of the greater hyoid’s horn, therefore a hyoid level of origin. It had a rectilinear antero-superior course reaching 0.74 cm superior to the greater horn, which gave off the LA and FA. The origin of the left LFT from the ECA was at 0.35 cm postero-infero-lateral to the tip of the greater horn. It ascended supero-medially and divided at 0.6 cm postero-superior to the tip of the greater horn.

**Figure 13:**
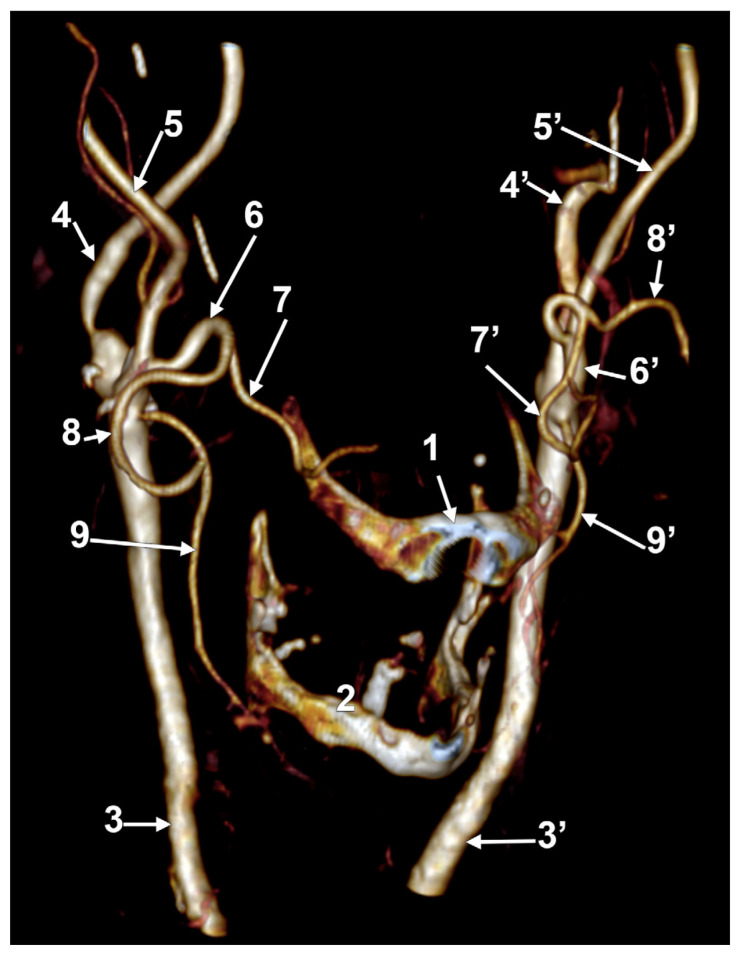
Bilateral linguofacial trunk. Computed tomography angiogram. Three-dimensional volume rendering. Right antero-inferior view. 1. hyoid body; 2. thyroid cartilage; 3, 3’. common carotid arteries; 4, 4’. internal carotid arteries; 5, 5’. external carotid arteries; 6, 6’. linguofacial trunks; 7, 7’. lingual arteries; 8, 8’. facial arteries; and 9, 9’. superior thyroid arteries.

In case #28 (male), the LFTs were asymmetrical (Figure 13). The right LFT originated at the hyoid bone level at 1.59 cm postero-lateral to the tip of its greater horn. It divided at 1.78 cm postero-supero-lateral to the greater horn into the LA and FA. The left LFT originated from the ECA in the interval between the hyoid and the gonial angle, at 1.25 cm postero-supero-lateral to the tip of the greater horn. It had a medial loop and divided at 1.8 cm postero-supero-lateral to the tip of the greater horn into the LA and FA.

**Figure 14:**
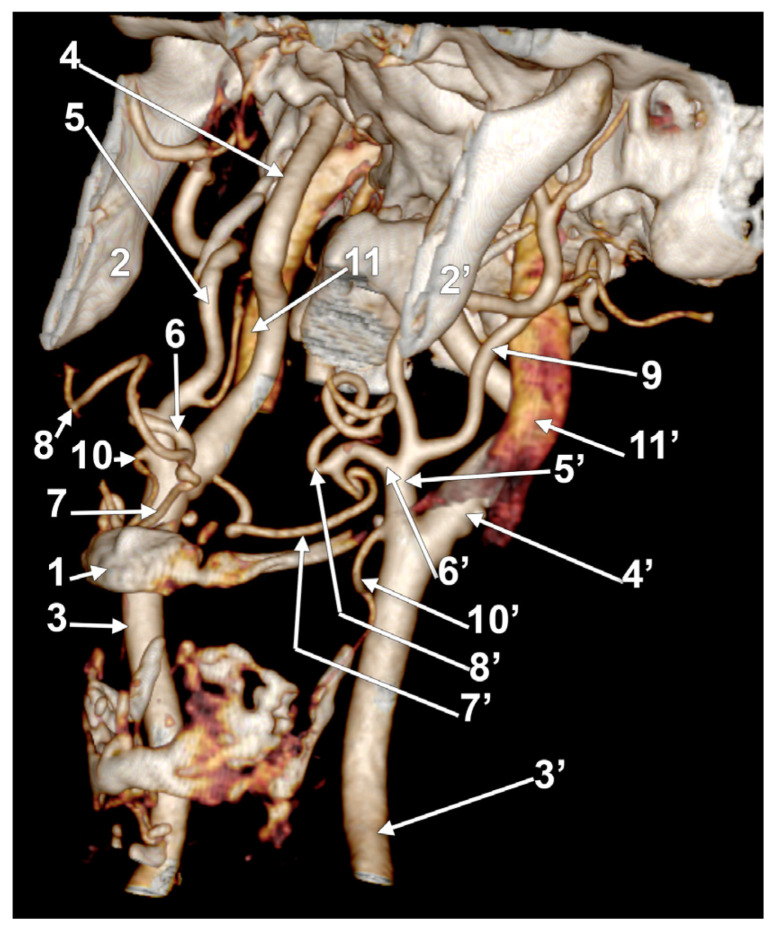
Bilateral linguofacial trunk. Computed tomography angiogram. Three-dimensional volume rendering. Left inferior view. 1. hyoid body; 2, 2’. gonial angles; 3, 3’. common carotid arteries; 4, 4’. internal carotid arteries; 5, 5’. external carotid arteries; 6, 6’. linguofacial trunks; 7, 7’. lingual arteries; 8, 8’. facial arteries; 9. left occipital artery; 10, 10’. superior thyroid arteries; and 11, 11’. internal jugular veins.

In case #29 (female), the right LFT left from the anterior side of a lateralized ECA between the hyoid and the gonial angle (Figure 14). Its origin was at 1.93 cm postero-supero-lateral to the tip of the greater hyoid horn. The proximal part of the right LFT looped anteriorly in front of the ECA, and its distal part looped posteriorly and reached 0.51 cm in front of the ICA. The right LFT divided into the LA and FA at 0.75 cm superior to the tip of the hyoid’s greater horn. The left ECA had a typical course, and the left LFT origin was from its anterior side, at 0.81 cm superior to the tip of the greater horn and inferior to the gonial angle. The left LFT had an anterior rectilinear course above the greater horn to divide into the LA and FA.

**Figure 15:**
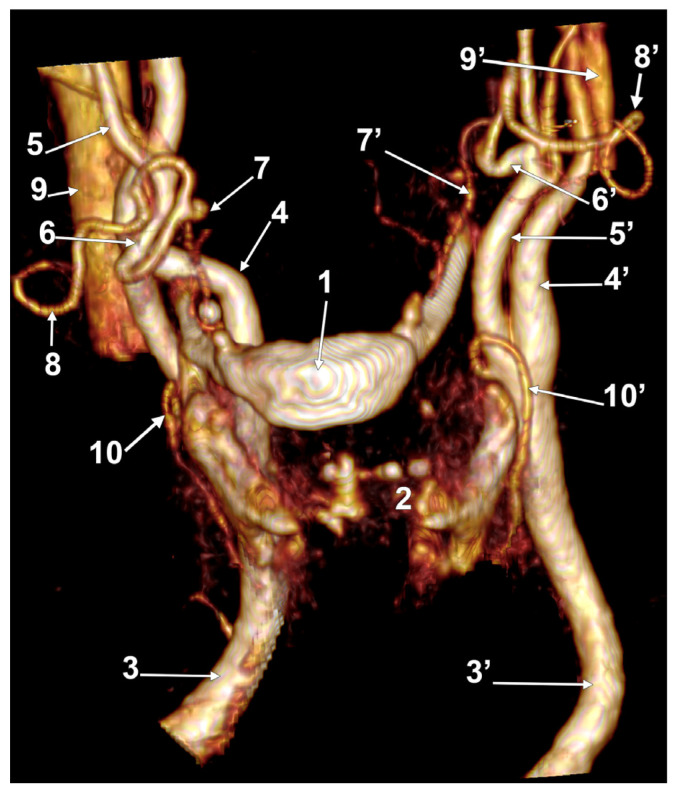
Bilateral linguofacial trunk. Computed tomography angiogram. Three-dimensional volume rendering. Left antero-lateral view. 1. hyoid body; 2. thyroid cartilage; 3, 3’. common carotid arteries; 4, 4’. internal carotid arteries; 5, 5’. external carotid arteries; 6, 6’. linguofacial trunks; 7, 7’. lingual arteries; 8, 8’. facial arteries; 9, 9’. internal jugular veins; and 10, 10’. superior thyroid arteries.

In case #30 (female), the right LFT left the ECA at 0.51 cm postero-superior to the tip of the greater horn, ascended supero-medially on the inner side of the ECA, and at 0.89 cm, supero-medial to the tip of the greater horn, divided into the LA and FA. The left LFT originated from the ECA at 0.98 cm supero-lateral to the tip of the greater hyoid horn; it was short (0.45 cm) and had a rectilinear course (Figure 15).

**Figure 16:**
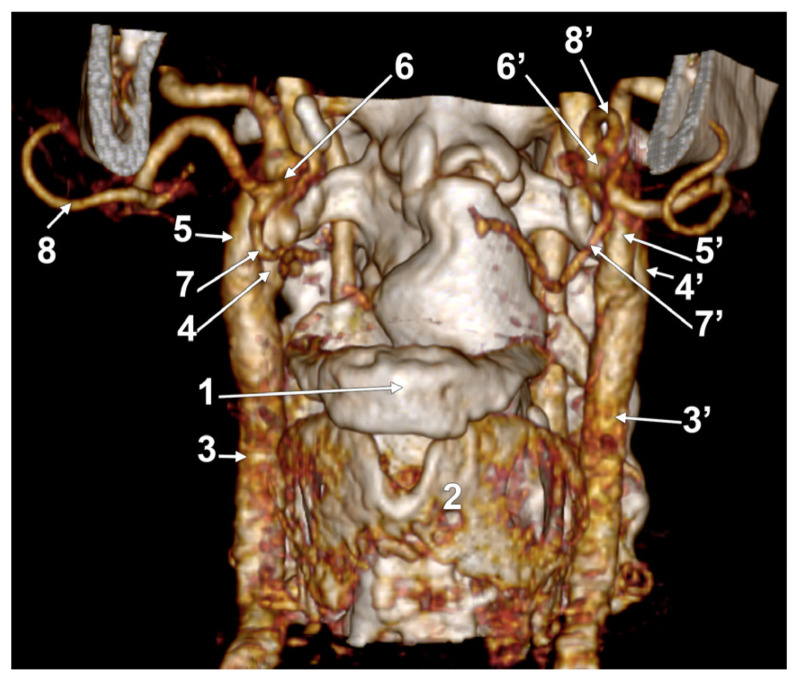
Bilateral linguofacial trunk. Computed tomography angiogram. Three-dimensional volume rendering. Left antero-lateral view. 1. hyoid body; 2. thyroid cartilage; 3, 3’. common carotid arteries; 4, 4’. internal carotid arteries; 5, 5’. external carotid arteries; 6, 6’. linguofacial trunks; 7, 7’. lingual arteries; and 8, 8’. facial arteries.

In case #31 (male), the left and right LFTs were short: the right was 0.77 cm, and the left was 0.26 cm. They were both rectilinear and had no loops (Figure 16).

**Figure 17:**
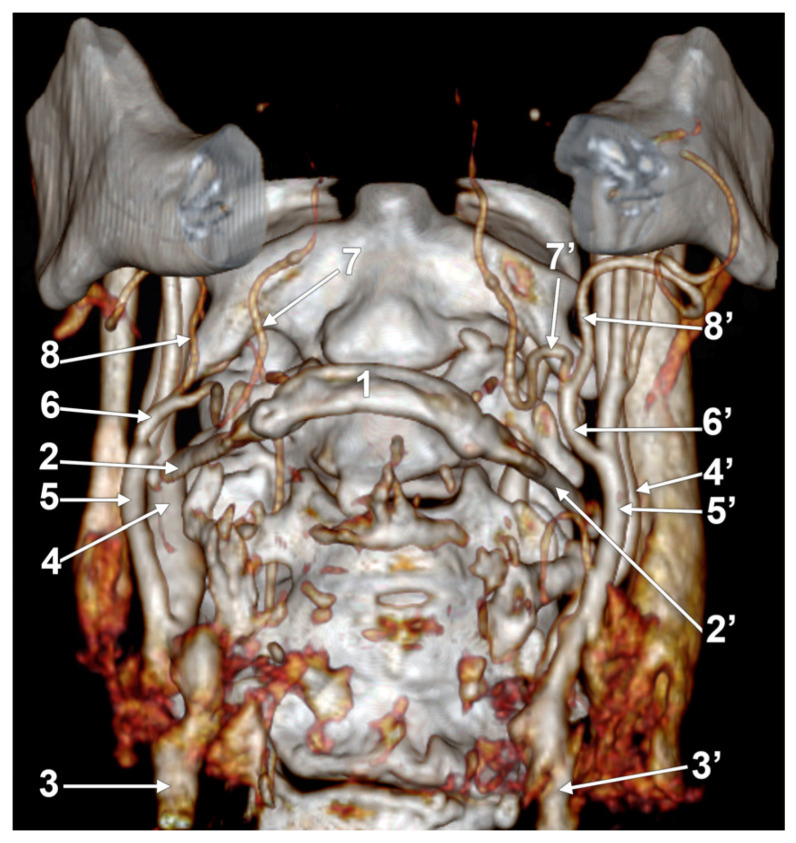
Bilateral linguofacial trunk. Computed tomography angiogram. Three-dimensional volume rendering. Antero-inferior view. 1. hyoid body; 2, 2’. greater horns of the hyoid bone; 3, 3’. common carotid arteries; 4, 4’. internal carotid arteries; 5, 5’. external carotid arteries; 6, 6’. linguofacial trunks; 7, 7’. lingual arteries; and 8, 8’. facial arteries.

Case #32 was a male one. Bilateral LFTs were found (Figure 17). On both sides, the LFTs originated in the interval between the hyoid bone and the gonial angle. On the right side, the LFT origin was at 0.29 cm superior to the greater horn’s tip, which, in turn, had an ossified triticeal cartilage medially. The rectilinear right LFT divided into the LA and FA at 0.95 cm superior to the greater horn. The left LFT origin was 0.46 cm superior to the hyoid’s greater horn and 0.85 cm antero-supero-lateral to its tip. At 1.15 cm, superior to the greater horn, it divided into the FA and LA.

**Figure 18:**
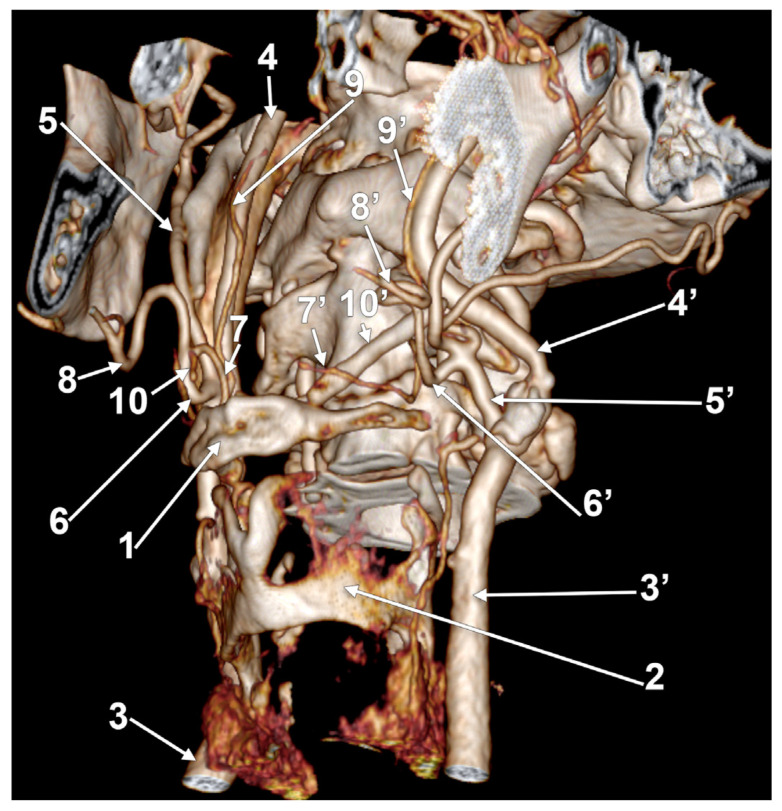
Bilateral linguofacial trunk. Computed tomography angiogram. Three-dimensional volume rendering. Left infero-lateral view. 1. hyoid body; 2. thyroid cartilage; 3, 3’. common carotid arteries; 4, 4’. internal carotid arteries; 5, 5’. external carotid arteries; 6, 6’. linguofacial trunks; 7, 7’. lingual arteries; 8, 8’. facial arteries; 9, 9’. ascending pharyngeal arteries; and 10, 10’. ossified ceratohyals.

Another male case (#33) had bilateral LFTs. Peculiarly, it had on the left side a stylohyoid chain that was almost entirely ossified (Figure 18, Video S1). On the right side, it had just a 0.75 cm long and 0.16 cm thick ceratohyal bone embedded within the stylohyoid ligament. The right LFT originated at 0.95 cm superior to the tip of the greater hyoid’s horn. It had an inferior loop approaching 0.93 cm above the greater horn. At 1.8 cm above it, the LFT divided into the LA and FA. Interestingly, the right LA had an initial posterior course, then redirected anteriorly and crossed the origin of the right LFT medially to reach above the greater horn.

On the opposite side, the stylohyoid chain was almost entirely ossified, but the inclinations of the stylohyal and ceratohyal differed. The left ceratohyal was laterally displacing three arteries (Figure 18). These were the occipital artery, the ECA, and the FA in a posterior-to-anterior sequence. The left occipital artery and LFT origins from the ECA were at the same level, resulting in a trident of the ECA. The left LFT had an inferior loop reaching 0.3 cm superior to the tip of the greater horn. At 0.92 cm above the greater horn, the LFT was divided into the LA and FA immediately inferior to the ossified ceratohyal. The resulting left LA looped medially inferior to the ceratohyal and continued above the greater horn but was on the lateral side of the arthrosis between the ceratohyal and hypohyal. The left FA ascended on the lateral side of the ceratohyal.

**Figure 19:**
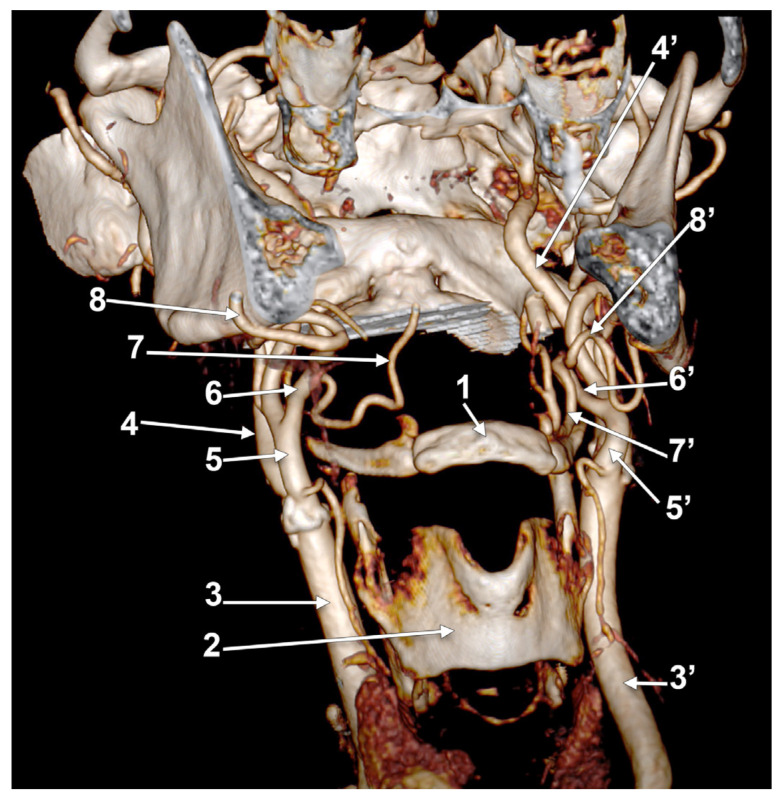
Bilateral linguofacial trunk. Computed tomography angiogram. Three-dimensional volume rendering. Right antero-infero-lateral view. 1. hyoid body; 2. thyroid cartilage; 3, 3’. common carotid arteries; 4, 4’. internal carotid arteries; 5, 5’. external carotid arteries; 6, 6’. linguofacial trunks; 7, 7’. lingual arteries; and 8, 8’. facial arteries.

The last case we present here, case #34, was a male case with bilateral LFTs (Figure 19). The right one’s origin was at 0.33 cm supero-lateral to the tip of the greater horn; it ascended supero-medially, with no loop. It divided into the LA and FA at 1.15 cm superior to the greater hyoid’s horn. Immediately posterior to the termination of the right LFT coursed the ascending pharyngeal artery. The left LFT origin from the ECA was at 0.48 cm supero-lateral to the greater hyoid horn; it coursed directly postero-supero-medially and divided into the LA and FA at 0.96 cm superior to the tip of the greater horn.

## 4. Discussion

Lippert and Pabst (1985) indicated an 18% prevalence of the LFT in their publication on arterial variations in man [2]. However, one could not find the size of the lots documented for that prevalence. Bergman’s Comprehensive Encyclopedia of Human Anatomic Variation mentions only that the FA and LA could have a common trunk of origin—the LFT [18]. However, the Illustrated Encyclopedia of Human Anatomic Variation by Bergman, Afifi, and Miyauchi mentions that the LA arises from a common trunk with the FA, the LFT, in 10–20% of cases [19]. The bilateral LFT is considered a rare variation [14]. Different dissection studies did not find bilateral LFTs [20,21]. A dissection study on 52 sides found LFTs only on the left side [22]. After a 30-cadaver study, no LFTs were detected [23]. In some previous studies, the LFT was termed the “faciolingual trunk” [24,25,26] or “lingual-facial trunk” [14,27].

The incidence of the LFT was variably reported, the unilateral cases being or not being distinguished from the bilateral ones (Table 1). Of these studies, a computed tomography study on 100 cases found a 15% prevalence of LFTs [28]. A different angioCT study on 76 cases found a prevalence of the LFT of 15.5%, but the uni- and bilateral cases were not distinguished [29]. The present CT study used 147 cases and found a 22.44% prevalence of the LFT.

A histological study revealed a thinner wall and a wider inner diameter of ECA, LA, and FA that originated from an LFT compared to those that originated separately [51].

Cappabianca et al. (2012) found different possibilities for the origin of the LFT: from the ECA (7%), from the CB (2%), or from the common carotid artery (1%) [7]. In a different study, only one from 34 LFTs left the CB, the others arising from the ECA [33]. In the present study, only ECA origins of the LFT were found. LFT crosses and obscures the ICA when it leaves a lateralized ECA [54]. A dissection study in 79 cadavers found more LFTs on the right side [62], but we couldn’t distinguish whether or not some of the reported LFTs were bilateral [62]. We found 47 LFTs, 13 unilateral left, eight unilateral right, and bilateral in the other 13 cases.

The insertion of an LFT into the ECA is also variable. In 56 dissected carotid systems, the sequence of ECA’s branch origins was the superior thyroid, LFT, occipital, and maxillary arteries in 21.4% and the superior thyroid, occipital, LFT, and maxillary arteries in 10.7% [26]. These patterns were considered to be due to a shift in the occipital artery branching site [26].

A recent dissection study in 35 cadavers was focused on the LA [63]. It was observed that the LFT tended to originate at a more superior level than a solitary LA [63]. The distance between the LFT and the greater horn of the hyoid bone was 1.59 + 0.52 cm on the right side and 1.67 + 0.61 on the left side [63]. Other authors determined the levels of origin for the solitary LA and, respectively, LFTs, as referred to the CBs [27]. They found no significant differences even though there was a tendency for the LFT to have a higher origin [27]. In 7/200 sides of fetuses, the only suprahyoid variant of the LA and FA origin from the ECA was the LFT [44].

A dissection study found the length of the LFT to be < 1 cm in 81.3% of sides and <1 cm in 18.7% of sides [26]. A CTA study found the mean length of the LFT to be 0.923 cm [29]. In a report of a unilateral LFT in a 103-year-old cadaver, the LFT was found to have a length of 0.88 cm [40]. Another study found the LFT to have an average length of 0.94 ± 0.17 cm on the right and 0.76 ± 0.13 cm on the left, with no difference between sides [27]. The present results do not support such a bilateral symmetry.

Recently, a short LFT further divided into the LA and FA was reported as an aberrant origin of the FA from the LA because the latter continued the course of the LFT [48]. An origin of the LA from the FA was also described (23.1% in 80 CTAs and 20% in 10 dissected cadavers) [64]. However, in our view, a common arterial trunk is just a common trunk, no matter the topography of its resulting branches.

Both the LFT and LA have individually variable loops. In 84% of cases, the LA forms a first superior loop with a variable height; in 16% of cases, the LA without the first superior loop runs forward obliquely; and in 8%, the LA forms an inferior loop [53]. Two subtypes of the LA’s superior loop were found: a double hump and an anticlockwise loop [53].

In case #9, the LA resulted from an LFT crossed over the FA. When the two arteries leave the ECA separately, such arterial crossing cannot occur. However, this possibility should not be ignored by surgeons because it can occur in cases with an LFT.

In case #12, an LFT was applied on the medial side of the ECA. When the ECA ligation is intended to spare the LA and FA territories, such an LFT should be distinguished and isolated.

The CB has different anatomical variables, such as the axial spin, the vertical location, and the hyoid–carotid topographical relations [9,65,66,67]. These modify the topography of the ECA’s branches [15]. Therefore, the anatomical variability of the morphology and topography of the LFT should be expected. As it was observed in this case-by-case study, the variables of the LFT are the ECA’s side of the origin of the LFT, the vertical height of the LFT origin (infrahyoid, hyoid, suprahyoid, subgonial), the morphology of the LFT (rectilinear, looped, coiled), and the length of the LFT. The potential loops of an LFT are diverse and thus unpredictable.

In case #10, a submandibular gland was displaced inferiorly over the hyoid bone and the ECA. Such submandibular gland ptosis and glandular enlargement obscure the ECA’s branches and complicate the identification of potential common arterial trunks. The submandibular gland ptosis is a significant contributor to age-related submandibular fullness [68].

Different reports of retropharyngeal carotid arteries are available. Most of these present retropharyngeal ICAs [69,70,71,72,73]. However, all carotid arteries could course medially to the greater horn of the hyoid bone [66], and are therefore posterior to the pyriform sinus. As it results from the present study (case #15), the LFT could also be retropharyngeal.

The stylohyoid chain derives from the second branchial arch. It is a bone–ligament complex consisting of the styloid process, the stylohyoid ligament, and the lesser horn of the hyoid bone [74]. It consists of four structures: the tympanohyal, forming the root of the styloid process; the stylohyal, forming the body of the styloid process; the ceratohyal, becoming the stylohyoid ligament; and the hypohyal, forming the lesser horn of the hyoid bone [74].

In case #33, we found asymmetrical bilateral ossifications of the stylohyoid chain. On one side, the ceratohyal was displacing the occipital artery, the ECA, and the FA laterally. Conversely, the ossified ceratohyal interfered with the LFT and the resulting FA. Therefore, different patterns of ossification of the stylohyoid chain could alter the normal course of different arteries and, eventually, determine an Eagle’s syndrome. These individual modifications could be easily documented on CTAs.

Arteries beneath the gonial angle are exposed and at risk during tonsillectomies. As we presented, the TLF could ascend and approach the tonsillar bed. Pseudoaneurysms may arise after a localized arterial laceration caused by blunt or penetrating trauma, including the traction and thermal damage produced by electrocautery [75]. Post-tonsillectomy hemorrhages from pseudoaneurysms of the LA, FA, ICA, and LFT were reported [75].

Although pseudoaneurysms of the LFT after tonsillectomy are rare, prompt diagnosis and timely management are mandatory to avoid profuse bleeding and death [76]. It can present intraoperatively and hours or days after tonsillectomy, either with massive bleeding or cervical swelling [76].

Superselective intra-arterial chemotherapy for oral cancer delivers a good concentration of anticancer agents into a tumor-feeding artery [77]. Such distribution through an LFT was investigated using computational fluid dynamics [77]. It was found that the agent’s behavior in the LFT is determined by the blood flow field, which depends on the topography of the vessels in each patient [77]. The latter was demonstrated here as an individual variable; therefore, it should be investigated before injections into the LFT. The catheter tip position should be changed according to the vessel topography to deliver anticancer agents into the tumor-feeding artery [77], the LA, or the FA. Nevertheless, vascular tortuosity, such as a loop or coil of an LFT, is expected to cause difficulties in catheter insertion [26].

## 5. Conclusions

The morphology and topography of the LFT are individually specific and unpredictable. It should be documented case-by-case by surgeons on CT or MR angiograms.

## Figures and Tables

**Table 1:** Previous studies and reports of the linguofacial trunk (LFT) are listed chronologically [6,7,8,9,11,12,14,20,21,22,24,25,26,28,29,30,31,32,33,34,35,36,37,38,39,40,41,42,43,44,45,46,47,48,49,50,51,52,53,54,55,56,57,58,59,60,61,62,63]. D: dissection. CTA: computed tomography angiography. MRA: magnetic resonance angiography. A: angiography. Cor.: corrosion casts. CR: case report. CB: carotid bifurcation.

Study	Method of Study, Lot	Prevalence (%) of the LFT	Observations
Homze et al., 1997 [60]	D, 91 sides	4.39%	–
Shangkuan et al., 1998 [53]	Cor., 14 sides	20%	–
Shintani et al., 1999 [24]	D, 29 cadavers	31%	–
Zumre et al., 2005 [11]	D, 20 specimens	10% on each side	study in fetuses
Lohan et al., 2007 [52]	MRA, 46 ECAs	unilateral LFT: 13.05% bilateral LFT: 2.17%	–
Anu et al., 2007 [57]	D, 95 cadavers	N/A	–
Anangwe et al., 2008 [9]	D, 40 cadavers	7%	–
Ozgur et al., 2008 [8]	D, 20 cadavers	7.5%	variable distance between the LFT and the CB
Fazan et al., 2009 [27]	D, 81 sides	right LFT: 20% left LFT: 24% bilateral LFT: 4.9%	lengths of the LFTs and levels of their origins were determined
Nirmaladevi and Sruthi, 2010 [42]	D, CR	N/A	–
Sanjeev et al., 2010 [31]	D, N/A	18.92%	quoted by Devadas et al., 2018 [12]
Thwin et al., 2010 [56]	D, CR	N/A	
Kishve et al., 2011 [45]	D, CR	N/A	LFT, ECA, occipital artery, and ICA origins from the upper end of the CCA (quadrifurcated CCA)
Vinnakota et al., 2011 [46]	D, CR	N/A	a medial course of a unilateral LFT
Troupis et al., 2011 [39]	D, 30 sides	3.33%	unilateral right LFT
Yonenaga et al., 2011 [26]	D, 56 sides	25.0%	–
Cappabianca et al., 2012 [7]	CTA, MRA, 97 cases	10%	LFT from the ECA (7%), from the CB (2%), or the common carotid artery (1%),
Mata et al., 2012 [32]	D, 36 sides	19.9%	–
Desai et al., 2012 [47]	D, CR	N/A	bilateral LFTs
Yadav et al., 2012 [37]	D, 26 cadavers	bilateral LFT: 3.8% unilateral LFT: 15.38%	–
Sirasanaglanda et al., 2012 [49]	D, CR	N/A	the resulting FA traversed the submandibular gland
Ahmed et al., 2012 [51]	D, 30 cadavers	unilateral LFT: 24.38% bilateral LFT: 2.27%	associated histological study
Dnyanesh et al., 2013 [34]	D, CR	N/A	abstract only
Pantoja et al., 2014 [40]	D, CR	N/A	morphometric data were brought
Gupta and Agarwal, 2014 [21]	D, 60 sides	unilateral LFT: 6.67%	–
Troupis et al., 2015 [14]	D, CR	N/A	bilateral LFTs
Kirchgessner, 2015 [54]	D, CR	N/A	LFT origin from a lateralized ECA
Heltzel et al., 2015 [62]	D, 79 cadavers	21.01%	–
Ogeng’o et al., 2016 [35]	D, 112 cadavers	44.7%	–
Kala et al., 2016 [44]	D, 200 sides	3.5%	study in fetuses
Ovhal et al., 2016 [20]	D, 120 sides	right LFT: 28.33% left LFT: 30%	–
Haldar et al., 2017 [30]	D, CR	N/A	Abs.
Anuradha and Chitra, 2017 [61]	D, 60 sides	unilateral LFT: 5%	LFT origin at 1.3 cm from the CB
Diwan et al., 2017 [43]	D, CR	N/A	unilateral LFT of 1.7 mm
Esakkiammal et al., 2017 [22]	D, 52 sides	13.46% left LFT	–
Devadas et al., 2018 [12]	D, 40 cadavers	bilateral LFTs: 12.5% unilateral LFTs: 7.5%	–
Baxla et al., 2018 [5]	D, CR	N/A	bilateral LFT
Paramasivam, 2019 [50]	D, CR	N/A	right LFT originating at 1.4 cm distally to the CB
Kumar and Kumar, 2019 [38]	D, CR	N/A	ascending LFT
Tippireddy et al., 2019 [23]			–
Herrera-Núñez et al., 2020 [29]	CTA, 152 sides	14.5%	uni- or bilateral LFTs were not distinguished
Cobiella et al., 2021 [33]	D, 207 sides	16.4%	1/34 LFTs from the CB, 33/34 LFTs from the ECA
Sudhakaran et al., 2021 [55]	D, 22 cadavers	13.6%	–
Nayak and Shetty, 2021 [64]			–
Charles et al., 2021 [58]	D, 30 sides	26.67%	–
Sarna et al., 2022 [63]	D, 35 cadavers	24.29%	–
Shreevastava et al., 2022 [41]	D, CR	N/A	bilateral LFTs with origin on different sides of the ECAs, anterior side on the right and medial side on the left
Rusu et al., 2022 [6]	CTA, CR	N/A	unilateral LFT
Kısaoğlu et al., 2022 [25]	D, CR	N/A	–
Sasikumar et al., 2023 [28]	CT, 100 cases	15%	–
Piagkou et al., 2023 [48]	D, CR	N/A	bilateral LFT a different LFT case was regarded as the aberrant origin of the FA from the LA
Gwunireama et al., 2023 [59]	D, 20 sides	unilateral LFT: 8%	–

## Data Availability

No new data were created or analyzed in this study. Data sharing is not applicable to this article.

## Supplementary Materials

The following supporting information can be downloaded at: https://www.mdpi.com/article/10.3390/medicina60020291/s1, Video S1: Almost complete ossification of the left stylohyoid chain in Case #33.
